# Super stretchable hydrogel achieved by non-aggregated spherulites with diameters <5 nm

**DOI:** 10.1038/ncomms12095

**Published:** 2016-06-29

**Authors:** Guoxing Sun, Zongjin Li, Rui Liang, Lu-Tao Weng, Lina Zhang

**Affiliations:** 1 Department of Civil and Environmental Engineering, The Hong Kong University of Science and Technology, Clear Water Bay, Hong Kong 999077, China; 2 Materials Characterization and Preparation Facility, The Hong Kong University of Science and Technology, Clear Water Bay, Hong Kong 999077, China; 3 Department of Chemical and Biomolecular Engineering, The Hong Kong University of Science and Technology, Clear Water Bay, Hong Kong 999077, China; 4 School of Material Science and Engineering, University of Jinan, 336, West Road of Nan Xinzhuang, Jinan 250022, Shandong, China

## Abstract

The scope of hydrogel applications can be greatly expanded by the improvement of mechanical properties. However, enhancement of nanocomposite hydrogels (NC gels) has been severely limited because the size of crosslinking nanoparticles is too large, at least in one dimension. Here we report a new strategy to synthesize non-aggregated spherulite nanoparticles, with diameters <5 nm, in aqueous solution, and their enhancement to hydrogel. The stress and stretch ratio at rupture of our NC gel are 430 and 121 KPa with only 40-p.p.m. nanoparticle content. The NC gel containing 200-p.p.m. nanoparticles can revert to 90% of its original size after enduring 100-MPa compressive stress. Our results demonstrate that the suppression of nanoparticle size without aggregation helps to establish a super stretchable and high-toughness hydrogel network at very low inorganic content.

## Abstract (web summary)

Hydrogels containing nanoparticles have enhanced mechanical properties that can be limited if the size of the crosslinking nanoparticles is too large. Here, Zhang and others have produced non-aggregated spherulite nanoparticles with diameters of less than 5 nm and investigated their effect on hydrogels.

## Graphical abstract

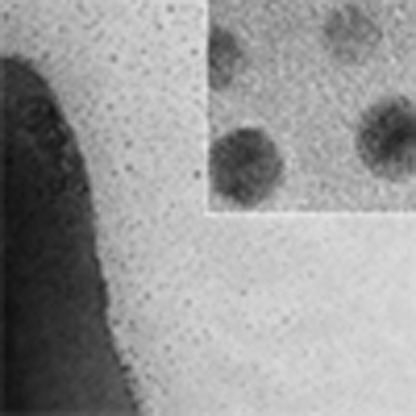

The mixing of nanoparticles with polymers to form composite materials has been practiced for decades. Nanoscale particles usually aggregate, and the aggregation becomes more serious with the decrease of particle size, and negates any benefits associated with the nanoscopic dimension^1^. There is a critical need, especially for nanocomposite hydrogels (NC gels), for non-aggregated nanoparticles at single-digit nanometre scale in an aqueous solution. Hydrogels can be used as scaffolds for tissue engineering^2^, temporary supports for cells^3^ and vehicles for drug delivery systems^4^. Conventional polymeric hydrogels usually exhibit weak strength and low toughness, and NC gels have been developed to improve the mechanical properties. In NC gels, polymer chains crosslink on the surface of the nanoparticles to establish a strong network, thus the mechanical properties of the hydrogel can be enhanced. However, either a large surface area or aggregation of the nanoparticles leads to uneven dispersion of the crosslink points, which greatly limits the property enhancement of the NC gels. In literature, the sizes of reported crosslinking nanoparticles were unfortunately all >60 nm at least in one dimension^5^,^6^,^7^,^8^,^9^,^10^,^11^,^12^,^13^,^14^,^15^,^16^,^17^,^18^. NC gel using inorganic clay particles was first reported in 2002 (ref. 5). Afterwards, various forms of sheet nanoparticles such as hectorite^6^,^7^,^8^,^9^, montmorillonite^10^, graphene oxide (GO)^11^,^12^,^13^,^14^,^15^, layered double hydroxide (LDH)^16^, titanate(IV) nanosheet^17^ and controllable activated nanogels^18^ were introduced to NC gels. With sizes of >800 nm in at least one dimension, clay has a concentration threshold (at least 1 wt%) for improving the strength of NC gels^5^,^6^,^7^,^8^,^9^. Such a high content of nanoparticles in the NC gels leads to residual and transparency problems. The incorporation of GO, which leads to the NC gels black and less translucent, makes the situation even worse^11^,^12^,^13^,^14^,^15^. Furthermore, the number of crosslink points in all these NC gels are restricted in the largest functional area (>2,800 nm^2^; Supplementary Table 1) of the particles, resulting in the aggregation of the crosslink points within the NC gel, which greatly limits the enhancement of mechanical properties. As a result, high stress, stretch ratio and recoverability are difficult to achieve at the same time in a typical NC gel. For example, hydrogel enhanced by exfoliated montmorillonite layers can be stretched up to 118 times of the initial length, but the maximum stress is limited to 100 KPa (ref. 10); and for hydrogels with a stress >200 KPa enhanced by high-content clay nanoparticles, the stretch ratio at rupture is <10 (ref. 6).

A more homogeneous distribution of high-density crosslink points is expected if the isotropic size of crosslinker nanoparticles can be suppressed to single-digit nanometres, without aggregation. In this work, we develop a new strategy to prepare calcium hydroxide (Ca(OH)_2_) nano-spherulites (CNS), with diameters <5 nm, which act as the crosslinker particles in polyacrylamide (PAM) hydrogel. The CNS separate individually without any aggregation, even after drying up or being implanted into the polymer matrix. The new PAM/CNS NC hydrogel system possesses many advantages over other NC hydrogels reported so far. For example, the PAM/CNS NC gel containing only 40-p.p.m. CNS has a swelling ratio of 250, a maximum stretch ratio of 121, and a stress up to 430 KPa, which are ∼5, 14 and 4 times higher than those of poly(*N*-isopropylacrylamide)/clay NC gel with 3 wt% clay^5^.

## Results

### Fabrication of CNS and PAM/CNS NC gels

**Figure 1:**
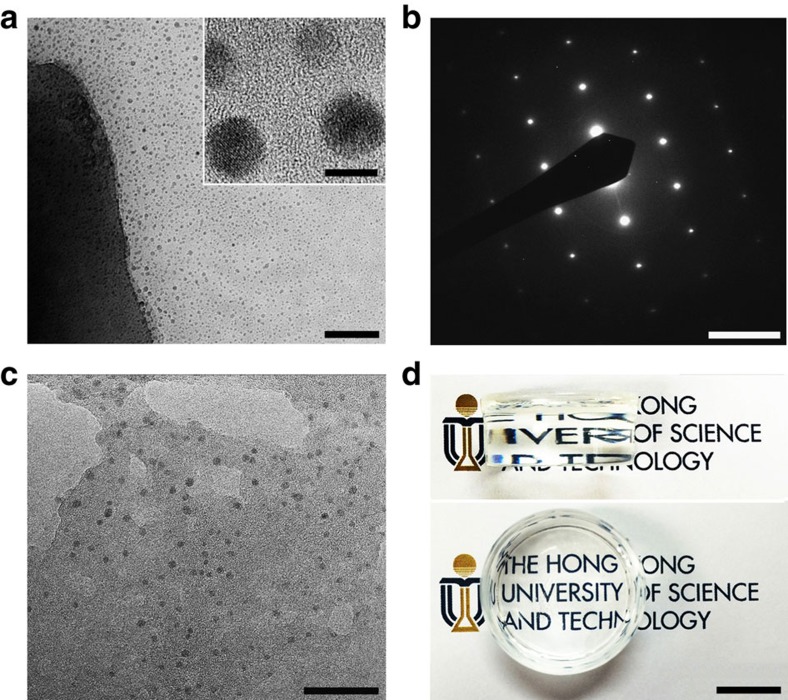
PAM enhanced by non-aggregated CNS. (**a**) TEM image of CNS released from a tricalcium silicate (Ca_3_SiO_5_) particle. The scale bars of the whole image and inset are 100 and 5 nm, respectively. (**b**) Electron diffraction patterns of CNS released from a hydrolysed Ca_3_SiO_5_ particle. Scale bar, 5 nm^−1^. (**c**) TEM image of CNS dispersed in C200 (PAM/CNS NC gel with 200 p.p.m. CNS concentration). Scale bar, 30 nm. (**d**) Optical photos of a cylindrical C200 specimen with a diameter of 2.7 cm and thickness of 1.2 cm against a background of characters. Scale bar, 1 cm. The transparency of C200 is almost the same as the original PAM hydrogel. PAM represents polyacrylamide; CNS represents calcium hydroxide nano-spherulites.

It has been reported that CNS of various sizes and small aggregates could be obtained in diols and water/oil microemulsions^19^,^20^. In this work, we used the hydration process of tricalcium silicate (Ca_3_SiO_5_), a main component of portland cement, to produce the CNS. When Ca_3_SiO_5_ particles are dispersed in water, calcium cations (Ca^2+^) are released from the Ca_3_SiO_5_ and form Ca(OH)_2_ crystals in the aqueous solution^21^. The hydrolysis temperature is critical in this process. We found that 0 °C was the optimal temperature because at this temperature, the releasing speed of calcium cations from the Ca_3_SiO_5_ was just enough to form Ca(OH)_2_ spherulites and at the same time the size of the spherulites was suppressed due to the low crystallization temperature. This can be clearly seen in Fig. 1a, which shows a transmission electron microscopy photograph taken on the surroundings of a hydrolysed Ca_3_SiO_5_ particle. The left side of Fig. 1a corresponds to a large block of Ca_3_SiO_5_ with a height of 500 nm, while the right side and the inset of Fig. 1a show the tiny spherulites with diameters 5 nm. Energy dispersive spectroscopy analysis confirms that these spherulites contain only Ca and O elements, while the electron diffraction pattern (Fig. 1b) and the observed lattice structure in the inset of Fig. 1a indicate that they are crystalline CNS. In aqueous media with a concentration of 200 p.p.m., CNS have a zeta potential of −10 mV screened by counterions (determined by dynamic light scattering testing) giving an electric double layer; these counterions ensure efficient dispersion of CNS at a concentration of 40–200 p.p.m. without any aggregation. It is anticipated that the controlled formation of CNS in an aqueous media would take place in hydrogel. PAM/CNS NC gels containing *n* p.p.m. CNS (named Cn as sample identification) were fabricated by *in situ* free-radical polymerization of acrylamide (AM) in suspension of CNS. As shown in Fig. 1c for C200 (sample with CNS 200 p.p.m. concentration), the dispersion of the spherulites with diameters >5 nm is indeed very homogeneous in the PAM matrix. Because of the low concentration, small size and homogeneous dispersion of the CNS, the transparency of C200 is almost the same as the original PAM hydrogel (Fig. 1d).

**Figure 2:**
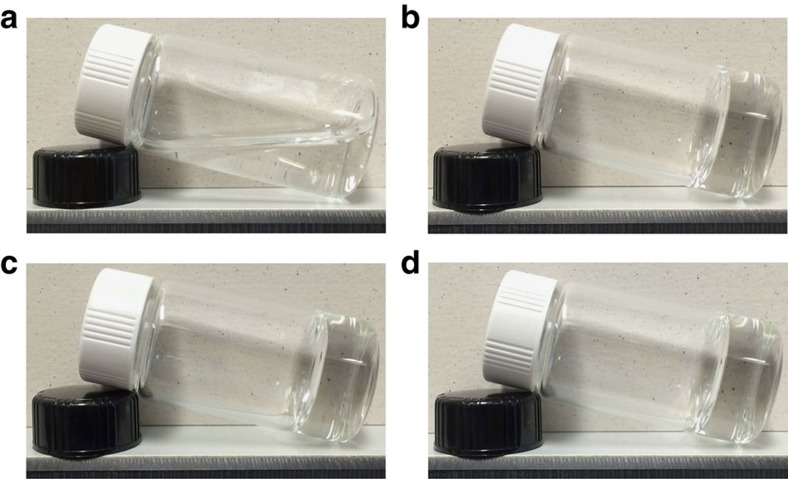
Gelation behaviour of PAM/CNS NC gels. The polymer and tricalcium silicate concentrations were fixed at 20 wt% and 500 p.p.m., respectively. (**a**) After being maintained at 0 °C and 1 atm for 10 days, gelation did not start at 1 atm. (**b**) After being maintained at 0 °C and 1 atm for 1 day, gelation started after 3 h at 0.01 atm. (**c**) After being maintained at 0 °C and 1 atm for 3 days, gelation started after 3 h at 0.01 atm. (**d**) After being maintained at 0 °C and 1 atm for 5 days, gelation started after 3 h at 0.01 atm. PAM/CNS NC gels represent polyacrylamide/calcium hydroxide nano-spherulites nanocomposite hydrogels.

**Figure 3:**
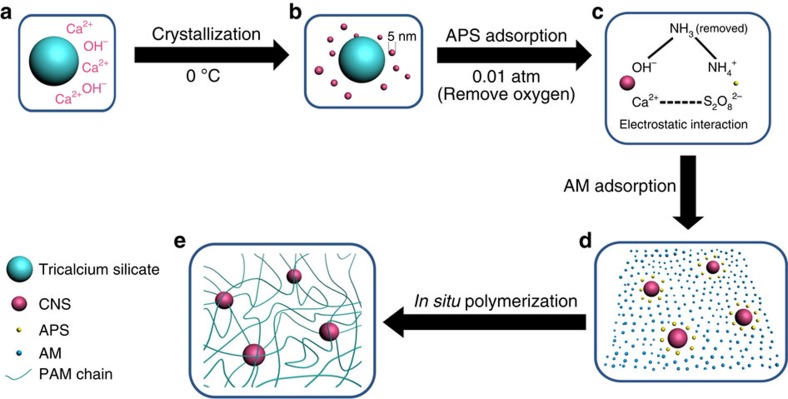
Schematic diagram of PAM crosslinked with the CNS released from tricalcium silicate (Ca_3_SiO_5_). (**a**) Ca^2+^ and OH^−^ released from the surface of a Ca_3_SiO_5_ particle during the hydration process. (**b**) CNS with diameters <5 nm formed from the crystallization of calcium hydroxide at 0 °C. (**c**) S_2_O_8_^2−^ from the initiator APS adsorbed on the surfaces of CNS via electrostatic interaction prompted by low pressure (0.01 atm). (**d**) Introduction of the monomer acrylamide (AM). (**e**) *In situ* polymerization of PAM on the surfaces of CNS.

The link between the PAM functional groups and CNS is established by acid–base reaction between the initiator ammonium peroxydisulfate (APS) and the Ca(OH)_2_. At room temperature, S_2_O_8_^2−^ (initiator anion) can be reduced by OH^−^ in the Ca_3_SiO_5_ suspension, thus polymerization is difficult to be initiated (Supplementary Fig. 1). However, at 0 °C, redox between S_2_O_8_^2−^ and OH^−^ is inhibited; the excess OH^−^ can react with NH_4_^+^ (initiator cation) to form gaseous NH_3_, which can be removed by a vacuum pump. Moreover, oxygen in the system can also be removed by the pumping process. Therefore, polymerization can start at 0 °C under vacuum conditions (0.01 atm) (Fig. 2). The whole fabrication procedure for the CNS, followed by the PAM/CNS NC gel, is shown in the schematic diagram (Fig. 3).

**Figure 4:**
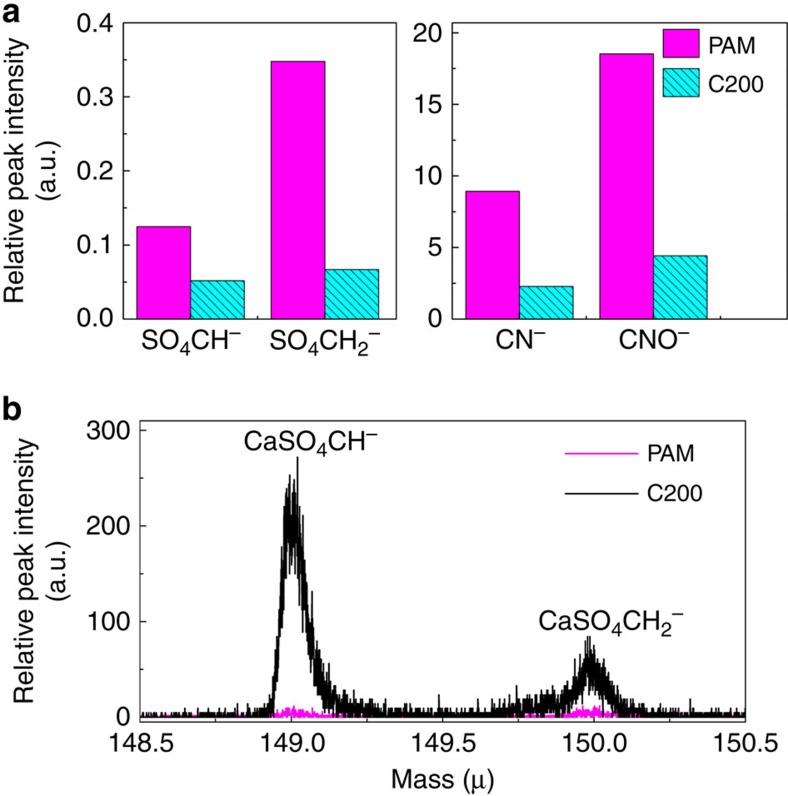
ToF-SIMS tests of PAM and C200. (**a**) Relative peak intensity of the negative ions representing the end (SO_4_CH^−^ and SO_4_CH_2_^−^) and side (CN^−^ and CNO^−^) functional groups of the original PAM and C200, with CH^−^ as the reference. The decreased relative peak intensity of the ions shows the reduction of the end and side groups in C200. (**b**) Peaks of CaSO_4_CH^−^ (mass 149.0) and CaSO_4_CH_2_^−^ (mass 150.0) appear in C200, and their relative intensities do not change before and after being stretched 60 times, indicating the strong connection between CNS and PAM end group. C200 represents PAM/CNS NC gel with 200 p.p.m. CNS concentration.

The connection between CNS and the PAM end group is determined, as being set-up by the ionic bonds between Ca^2+^ and S_2_O_8_^2−^. To prove this mechanism, time-of-flight secondary ion mass spectrometry (ToF-SIMS) is employed to get the characteristic ion peaks of the original PAM and C200. With the unique PAM backbone ion CH^−^ as reference, the relative peak areas of the negative ions SO_4_CH^−^ and SO_4_CH_2_^−^ are chosen to represent end groups in the PAM chains, while CN^−^ and CNO^−^ represent the side groups. Compared with the original PAM, the decreased relative peak intensity of SO_4_CH^−^ and SO_4_CH_2_^−^ shows the reduction of the end groups in C200 (Fig. 4a). Similarly, the side group –CONH_2_ can react with OH^−^ to form gaseous NH_3_, which can be promoted by vacuum, and hydrolysed to –COO^−^, which then connects with Ca^2+^ on the surface of CNS. Such a reaction pathway is evidenced from the decrease of the relative peak intensity of CN^−^ and CNO^−^ (Fig. 4a). The appearance of the peaks of CaSO_4_CH^−^ and CaSO_4_CH_2_^−^ in C200 strongly supports the chemical bonding between CNS and the PAM end groups (Fig. 4b). Moreover, their relative intensities do not change before and after being stretched for 60 times, indicating the strong connection between CNS and the PAM end group.

### Crosslinked network of PAM/CNS NC gels

The weight of a piece of freeze-dried xerogel of C40 (sample with CNS 40 p.p.m. concentration), before and after swelling in a large excess of deionized water for 3 weeks, shows that <0.1 wt% weight is lost, which implies a complete crosslinked network formation of the NC gel with only 40 p.p.m. CNS. At the same volume content and dispensability, the particle number density of the CNS with diameters <5 nm is ∼1,000, 2,000, 200 and 5.0 × 10^5^ times higher than that of hectorite, GO, LDH and titanate(IV) nanosheet, respectively (Supplementary Table 1). The crosslink points disperse much more homogeneously on the surfaces of CNS with the surface area <78.5 nm^2^ compared with that of nanosheets with functional surface area >2800, nm^2^ (Supplementary Table 1). The distribution and density of the crosslink points are very important in the establishment of the crosslinked network. For example, we obtained Ca(OH)_2_ micro crystals by cooling a suspension of Ca(OH)_2_ from room temperature to 0 °C (Supplementary Fig. 2). PAM-based hydrogel named CA200 (sample with Ca(OH)_2_ micro crystal 200 p.p.m. concentration) was fabricated in the same way as the PAM/CNS NC gel. At the same Ca(OH)_2_ weight content, the number density of the crosslink points in CA200 is only 1/3.8 × 10^6^ compared with that in the NC gel (Supplementary Table 1). Such low crosslink density makes a poor network of CA200, which has very limited mechanical enhancement and does not swell, but degenerates and dissolves in water.

**Figure 5:**
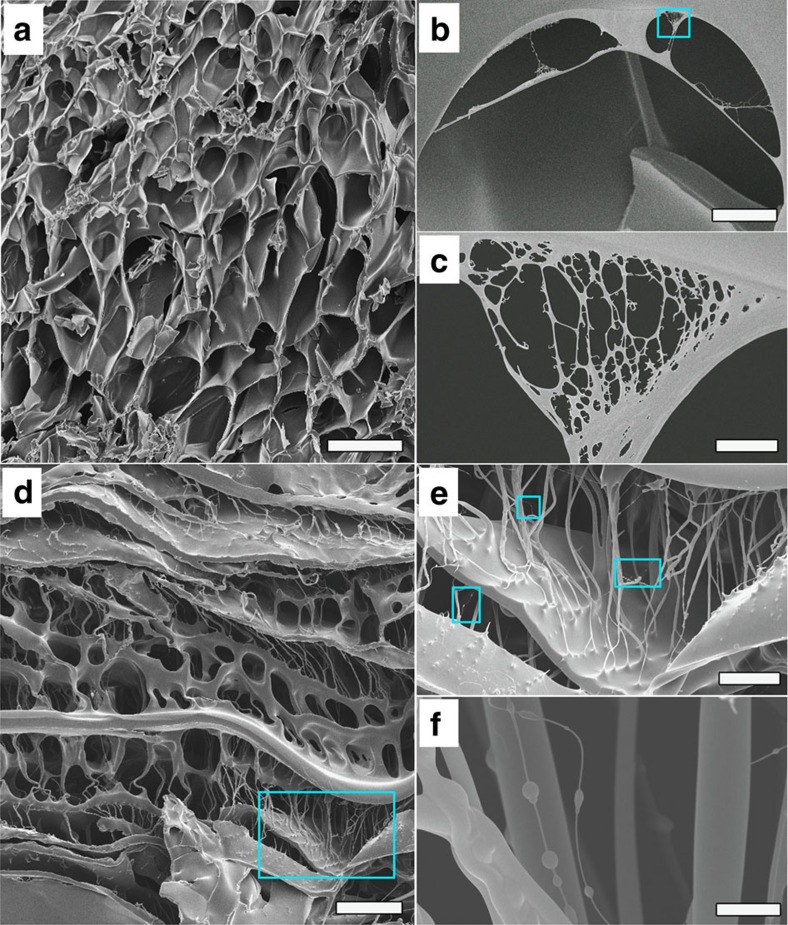
Morphology and structure of PAM/CNS xerogels. (**a**) Scanning electron microscope (SEM) image of C40 (NC gel with 40 p.p.m. CNS concentration) xerogel. Scale bar, 500 μm. (**b**) Zoomed SEM image of the box region in **a**. Scale bar, 30 μm. (**c**) Zoomed SEM image of the box region in **b**. Scale bar, 2 μm. (**d**) SEM image of C200 (NC gel with 200 p.p.m. CNS concentration) xerogel. Scale bar, 100 μm. (**e**) Zoomed SEM image of the box region in **d**. Scale bar, 30 μm. (**f**) Zoomed SEM image in the region of the top one of the three boxes in **e**. Scale bar, 2 μm.

Because of the homogeneous distribution and high number density of the CNS particles, the crosslinked network of the NC gel can be effectively controlled by the CNS concentration. Suspension of CNS with concentration 40 p.p.m. (determined by inductively coupled plasma mass spectrometry) can be obtained by dispersing 100 p.p.m. Ca_3_SiO_5_ in water at 0 °C for 3 days. The maximum concentration of CNS is 200 p.p.m., obtained by dispersing 500 p.p.m. Ca_3_SiO_5_ in water at 0 °C for 3 days. This concentration will not increase even with higher Ca_3_SiO_5_ content or longer hydration time, because of the upper limit of calcium cation (Ca^2+^) concentration in Ca_3_SiO_5_ suspension^21^. Figure 5a–f present typical s.e.m. images, showing the morphologies of the freeze-dried samples of C40 and C200 xerogels. Differing from the nanosheet-reinforced NC gels, such as the PAM/LDH NC gel that has a hierarchical porous morphology with an irregular distribution of micro- and nanometre scale pores^16^, the freeze-dried xerogel of C40 demonstrates a uniform distribution of porous structures with diameters of 200–400 μm (Fig. 5a). Interconnected nanopores can be observed in the inner walls of the micrometre-size pores (Fig. 5b). Moreover, Fig. 5c further reveals that the diameters of most of these nanopores are <300 nm, indicating the crosslink points disperse homogeneously at the nanometre scale. With an increased crosslinker concentration, the diameters of the porous structures in the xerogel of C200 are smaller than those of C40, ∼20–80 μm, and a great many parallel and crosslinked filamentous structures, with diameters from 50 nm to 2 μm, are distributed regularly inside the pores (Fig. 5d). The obvious embossments at the connection point between the filaments and inner walls of the pores demonstrate the strong connection force (Fig. 5e), which plays an important role in the swelling and mechanical properties of the NC gel.

**Figure 6:**
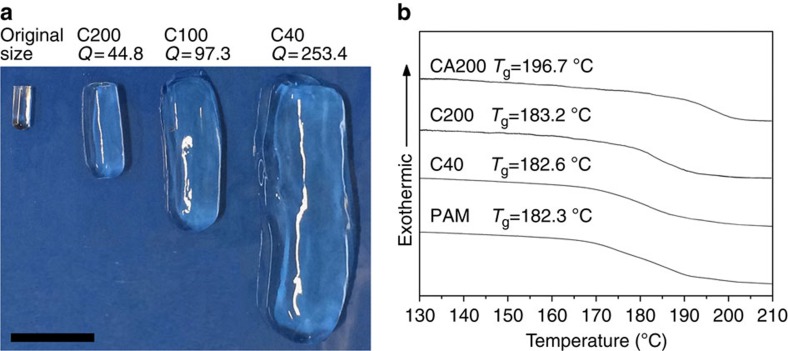
Mobility and flexibility of the polymer molecules for PAM/CNS NC gels. (**a**) An optical photo of swollen hydrogels of C40, C100 and C200 after soaking at a large excess of deionized water for 3 weeks. Scale bar, 60 mm. (**b**) DSC curves showing that the values of *T*_g_ for C40 and C200 are slightly higher than that for the neat PAM, and the *T*_g_ of CA200 is obviously higher than the others.

Compared with C200, the crosslinked network of C40 has a bigger swelling capacity because there are few connecting filaments and larger pore size. With the original shape maintained, the swelling ratio (*Q*) of C40 is 253.4, ∼2.6 and 5.7 times higher than that of C100 (sample with CNS 100 p.p.m. concentration, obtained by dispersing 250 p.p.m. Ca_3_SiO_5_ in water at 0 °C for 3 days) and of C200 (Fig. 6a). So, a large range of *Q* values from 44.8 to 253.4 can be obtained by simply adjusting the concentration of CNS in the NC gels. Unique shish-kebab structures can be observed on the filaments with diameters ∼50 nm in the micrometre pores of the C200 xerogels (Fig. 5f and box regions in Fig. 5e). We believe that the bumps with diameters of 100–500 nm are CNS coated with PAM chains. The thickness of the coating layer (100–500 nm) and the distance between the bumps (700 nm–3 μm) suggest that the introduction of CNS has a limited confinement effect on the mobility and flexibility of the polymer molecules for the present NC gels. Another piece of evidence is provided by the glass transition temperature (*T*_g_), determined by differential scanning calorimeter measurement (Fig. 6b). Our results show that the *T*_g_ of the NC gels (C40: 182.6 °C and C200: 183.2 °C) are slightly higher than that of linear PAM (182.3 °C) and almost independent of CNS concentration. In contrast, CA200 has an obvious increased *T*_g_ of 196.7 °C, indicating strong restricted force of larger size particles. Therefore, the polymer molecules in the crosslinked network of the NC gels are free, highly flexible and mobile, taking on nearly random conformations between the CNS.

### Mechanical properties of PAM/CNS NC gels

**Figure 7:**
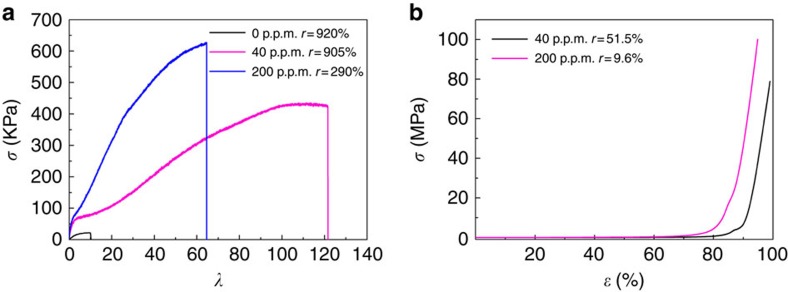
Elongation and compressive tests of PAM/CNS NC gels at 25 °C. (**a**) Representative elongation stress–strain curves of PAM hydrogels containing 0, 40 and 200 p.p.m. CNS. *σ* represents the stress. *λ* represents the stretch ratio. *r* represents the ratio of the length change along the direction of load after the load is released with respect to the original length of the specimen. (**b**) Compression stress–strain curves of C40 (NC gel with 40 p.p.m. CNS concentration) and C200 (NC gel with 200 p.p.m. CNS concentration). *ɛ* represents the strain.

The mechanical properties of hydrogels are strongly affected by the crosslinked network structure controlled by the concentration of CNS (Fig. 5a–f). To establish the relationship between the network structure and the mechanical properties, we performed elongation tests on the NC gels with different CNS concentrations at room temperature. The representative stress–elongation curves showed that the maximum stress of PAM-based hydrogel increased from 20 to 430 KPa, and the stretch ratio at rupture increased from 10 to 121 (Fig. 7a; Supplementary Movie 1), with the addition of only 40 p.p.m. CNS. The residual deformation ratio (*r*), which is defined as a ratio of the length change along the direction of load after the load is released with respect to the original length of the specimen, can be considered as an index of recoverability. After being stretched 121 times, the specimen C40 could revert to 905% of its initial length, indicating good recoverability. The stretch ratio at rupture of C40 is higher than the reported highest value for NC gel^10^, and the maximum stress is more than four times higher at the same time (c.f. Table 1). The fracture toughness of C40 reaches up to 33.9 MJ m^−3^ because it can achieve high stress and stretch at the same time. The noteworthy tensile properties of C40 can be explained by the following reason: non-aggregated CNS with diameters <5 nm provide homogeneous micro-pores in the crosslinked network, and the lower CNS concentration leads to fewer connecting filaments and larger pore size, which makes for easier deformation (Fig. 5a–c). The stress of C200 reaches 630 KPa at a rupture stretch ratio of 65 and a residual deformation ratio of 290% (Fig. 7a; Supplementary Movie 2), which is ∼2.7 times that of the reported highest value of the transparent PAM-based hydrogels with alginate (the stretch ratio is 16 times higher at the same time, c.f. Table 1)^22^. The fracture toughness of C200 is 26.2 MJ m^−3^, which is lower than that of C40, because of the decreased deformation ability of the crosslinked network. However, the smaller pore size and the establishment of the filament structure connecting the inner walls of the pores provide a strong mechanical strength and restoring force for C200 (Fig. 5d–f). In compressive testing, C200 almost reverts to its original size with a residual deformation ratio *r*=9.6%, after enduring a compressive stress of 100 MPa at strain of 95% (Fig. 7b; Supplementary Movie 3). In contrast, although C40 can endure a compressive stress of 78 MPa at strain of 99% without fracture, it only reverts to about half of its original size with a residual deformation ratio *r*=51.5% (Fig. 7b; Supplementary Movie 4).

**Figure 8:**
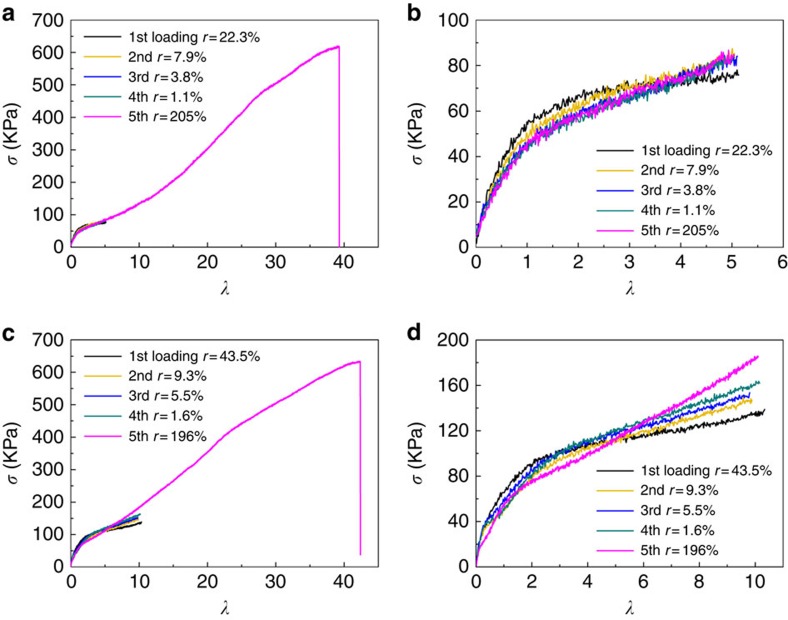
Cyclic elongation tests of PAM/CNS NC gels at 25 °C. (**a**) Cyclic elongation stress–strain curves of C200 (NC gel with 200 p.p.m. CNS concentration) elongated for five-fold in four cycles and elongated to rupture at the fifth cycle. (**b**) Enlarged cyclic elongation stress–strain curves of C200 elongated for five-fold in four cycles. (**c**) Cyclic elongation stress–strain curves of C200 elongated for 10-fold in four cycles and elongated to rupture at the fifth cycle. (**d**) Enlarged cyclic elongation stress–strain curves of C200 elongated for 10-fold in four cycles. *σ* represents the stress. *λ* represents the stretch ratio. *r* represents the ratio of the length change along the direction of load after the load is released with respect to the original length of the specimen.

The remarkable recoverability of C200 can be further evaluated by cyclic elongation tests (Fig. 8). When stretched five-fold from the first loading to the fifth loading, the cyclic elongation stress–strain curves of C200 nearly overlapped each other, indicating small hysteresis effects in the specimen. The residual deformation ratio *r* of the first cycle is 22.3%, and further decreases to 1.1% after being stretched over four cycles (Fig. 8a). The stretch ratio of the specimen in each cycle was calculated by the recovered length, with a slightly irreversible deformation after being stretched in the last cycle. So, the stretch ratio at rupture of the C200 specimen in the fifth cycle is ∼40 with a residual deformation ratio of 205% due to the previous irreversible deformation in the pre-cyclic elongation. The enlarged elongation stress–strain curves in Fig. 8b show that the tensile modulus of the first cycle is slightly higher than that of the other cycles when stretched less than four-fold, and decreases with further stretching. It demonstrates that during the initial elongation, there was a slightly irreversible deformation of the crosslinked network. Then, the remarkable recoverability of the NC gel was achieved after the initial conformation adjustment of the crosslinked network. The recoverability can also be observed by stretching the C200 NC gel 10-fold (Fig. 8c,d). Because the NC gel was cyclic stretched with a higher ratio in the first to fourth cycles, the residual deformation ratio *r* at each cycle was higher than that obtained in the five-fold cyclic tests. However, with a more sufficient conformation adjustment, the residual deformation ratio *r* in the fifth cycle (stretched to rupture) decreased to 196%. It demonstrates that good recoverability along the direction of the elongation load can be observed with an initial slight irreversible deformation.

## Discussion

The properties of the PAM/CNS NC hydrogels can thus be tuned through the concentration of CNS, which will give high values for swelling, extensibility, breakage stress and recoverability to the hydrogels. The non-aggregated CNS can also be obtained via a suspension of portland cement instead of Ca_3_SiO_5_ (Supplementary Fig. 3), and can be further applied for property enhancement in other polymeric hydrogels synthesized via *in situ* polymerization. Our results demonstrate that individually separated spherulites with diameters <5 nm can be obtained from the low-temperature hydration product of Ca_3_SiO_5_, and may help to establish a new NC gel system with enhanced crosslinked networks and properties.

## Methods

### Materials

AM, APS and *N*,*N*,*N*′,*N*′-tetramethyl-ethylenediamine (TEMED; 99%), were purchased from Sigma-Aldrich Chemical Reagent Co., Ltd, and used without further purification. All the chemicals, including calcium carbonate (Alfa Aesar) and silica (4 N, Sinopharm Chemical Reagent Co., Ltd) were of analytical pure reagent (A.R.) grade. In all the experiments, deionized water was used.

### Synthesis of Ca_3_SiO_5_

Calcium carbonate and silica (molar ratio of 3:1) were first ground to pass through a 63-μm sieve and then evenly mixed for 1–2 h. The obtained fine powder was compressed into pancakes in a lab press and put into a Pt crucible for calcination at 1,500 °C for 5 h, and quickly cooled down to ambient temperature within 10 min. The obtained product was ground into fine powder, compressed and calcined again. After repeating the above process for four cycles, the final Ca_3_SiO_5_ with particle size <500 nm was obtained (Supplementary Fig. 4).

### Preparation of (CNS) suspension

Ca_3_SiO_5_ was dispersed in deionized water to obtain a translucent aqueous dispersion, and the system was stirred under ultrasonication for 10 min to yield a homogeneous dispersion. The whole process proceeded at 0 °C in an ice bath.

### Preparation of PAM/CNS NC gels

The PAM/CNS NC gels were prepared by *in situ* free-radical polymerization. The monomer AM, the initiator APS and the accelerator TEMED were added into the CNS suspension of composition: CNS suspension/AM/APS/TEMED=60 g/15 g/0.03 g/48 μl. For the complete reaction of CNS and APS, the mixture was kept at 0 °C in an ice bath for at least 72 h. The polymerization process proceeded in a vacuum environment (0.01 atm) at 0 °C.

### Characterization of morphology and structure of the NC gels

The morphology and distribution of CNS were characterized by transmission electron microscopy (JEOL, JEM-2100, 200 kV), equipped with an energy dispersive spectroscopy system. The structure of the xerogels was characterized by a high-resolution scanning electron microscope (JEOL, model JSM-6700F). The *T*_g_ of the hydrogels were determined by a differential scanning calorimeter (TA Q1000). ToF-SIMS was used to demonstrate the existence of the PAM end and side groups, and CNS. Static ToF-SIMS spectra of pure PAM and NC gel C200 were obtained from a ToF-SIMS V spectrometer (ION-TOF GmbH, Münster, Germany). The samples were bombarded with Bi_3_^+^ primary ions, which were accelerated at 25 kV with an average pulsed current of 0.3 pA. The raster area was 200 × 200 μm, and the acquisition time for each spectrum was 40 s, corresponding to an ion dose of <4 × 10^11^ ions per cm^2^. Three positive and negative spectra were recorded for each specimen at different locations.

### Mechanical tests

Mechanical tests were conducted at 25 °C using a MTS (model E44, EXCEED) testing machine. The samples for the elongation tests were of rod-like shape 30 mm in length and 3.2 mm in diameter for the as-prepared PAM/CNS NC gels. The stretched portion of the samples between the two clamps was 2.0 mm for C40 and 1.8 mm for C200, and the samples were elongated at a loading rate of 50 mm min^−1^ (Supplementary Movies 1 and 2). For the compression tests, cylindrical samples of the as-prepared hydrogels were used, with dimensions of 11.8 mm in diameter and 6.1 mm in height for C40, and 10.3 mm in diameter and 5.7 mm in height for C200. The crosshead speed was 1 mm min^−1^, and stopped at the maximum loading of 8,400 N (Supplementary Movie 3 and 4).

### Swelling experiments

Swelling experiments were performed by immersing the as-prepared hydrogels in a large excess of deionized water at 25 °C for 3 weeks to reach swelling equilibrium. During this period, the water was replaced several times. The swollen samples were then freeze-dried. The swelling ratio (*Q*) was evaluated using *Q*=*W*_s_/*W*_d_, where *W*_s_ and *W*_d_ are the weights of the swollen sample and the corresponding dried xerogel sample, respectively.

### Data availability

The data that support the findings of this study are available from the corresponding author upon request.

## Additional information

**How to cite this article:** Sun, G. *et al*. Super stretchable hydrogel achieved by non-aggregated spherulites with diameters <5 nm. *Nat. Commun.* 7:12095 doi: 10.1038/ncomms12095 (2016).

## Supplementary Material

Supplementary Information Supplementary Figures 1-4 and Supplementary Table 1

Supplementary Movie 1 An elongation test of PAM/CNS NC gel with 40 ppm CNS concentration. The specimen is of rod-like shape with 30 mm in length and 3.2 mm in diameter. The stretched portion of the samples between the two clamps is 2.0 mm.

Supplementary Movie 2 An elongation test of PAM/CNS NC gel with 200 ppm CNS concentration. The specimen is of rod-like shape with 30 mm in length and 3.2 mm in diameter. The stretched portion of the samples between the two clamps is 1.8 mm

Supplementary Movie 3 A compressive test of PAM/CNS NC gel with 200 ppm CNS concentration. The specimen is of cylindrical shape with 10.3 mm in diameter and 5.7 mm in height.

Supplementary Movie 4 A compressive test of PAM/CNS NC gel with 40 ppm CNS concentration. The specimen is of cylindrical shape with 11.8 mm in diameter and 6.1 mm in height.

Peer review file

## Figures and Tables

**Table 1:** Elongation stress and stretch ratio at rupture of synthetic elastic hydrogels with different crosslinkers.

**Matrix**	**Particle**	**Content (p.p.m.)**	**Stress (KPa)**	**Stretch ratio**
Poly(*N*-isopropylacrylamide)	Hectorite^5^,^6^*	30,000	109	8
		125,000	1,000	10
Polyacrylamide	Montmorillonite^10^*	8,700	100	118
		80,800	175	85
	Graphene oxide^11^*	80	282	31
		480	385	34
	Layered double hydroxide^16^	8,000	34	62
	Alginate^22^	110,000	150	23
		330,000	230	4
	CNS (this work)	40	430	121
		200	630	65

CNS, calcium hydroxide nano-spherulites.

^*^Hydrogels containing high-content clay and graphene oxide have residual and transparency problems.

## References

[b1] Balazs A. C., Emrick T. & Russell T. P. Nanoparticle polymer composites: where two small worlds meet. Science 314, 1107–1110 (2006). 17110567 10.1126/science.1130557

[b2] Lee K. Y. & Mooney D. J. Hydrogels for tissue engineering. Chem. Rev. 101, 1869–1879 (2001). 11710233 10.1021/cr000108x

[b3] Nicodemus G. D. & Bryant S. J. Cell encapsulation in biodegradable hydrogels for tissue engineering applications. Tissue Eng. Pt B Rev. 14, 149–165 (2008). 10.1089/ten.teb.2007.0332 PMC2962861 18498217

[b4] Hoffman A. S. Hydrogels for biomedical applications. Adv. Drug Deliver. Rev. 54, 3–12 (2002). 10.1016/s0169-409x(01)00239-3 11755703

[b5] Haraguchi K. & Takehisa T. Nanocomposite hydrogels: a unique organic-inorganic network structure with extraordinary mechanical, optical, and swelling/de-swelling properties. Adv. Mater. 14, 1120–1124 (2002).

[b6] Haraguchi K. & Li H. J. Control of the coil-to-globule transition and ultrahigh mechanical properties of PNIPA in nanocomposite hydrogels. Angew. Chem. Int. Ed. Engl. 44, 6500–6504 (2005). 16158451 10.1002/anie.200502004

[b7] Haraguchi K., Ebato M. & Takehisa T. Polymer-clay nanocomposites exhibiting abnormal necking phenomena accompanied by extremely large reversible elongations and excellent transparency. Adv. Mater. 18, 2250–2254 (2006).

[b8] Haraguchi K., Li H. J., Matsuda K., Takehisa T. & Elliott E. Mechanism of forming organic/inorganic network structures during *in-situ* free-radical polymerization in PNIPA-clay nanocomposite hydrogels. Macromolecules 38, 3482–3490 (2005).

[b9] Haraguchi K. & Li H. J. Mechanical properties and structure of polymer-clay nanocomposite gels with high clay content. Macromolecules 39, 1898–1905 (2006).

[b10] Gao G., Du G., Sun Y. & Fu J. Self-healable, tough, and ultrastretchable nanocomposite hydrogels based on reversible polyacrylamide/montmorillonite adsorption. ACS Appl. Mater. Interfaces 7, 5029–5037 (2015). 25668063 10.1021/acsami.5b00704

[b11] Liu R. Q. . Tough and highly stretchable graphene oxide/polyacrylamide nanocomposite hydrogels. J. Mater. Chem. 22, 14160–14167 (2012).

[b12] Zhang N. N. . Actuator materials based on graphene oxide/polyacrylamide composite hydrogels prepared by in situ polymerization. Soft Matter 7, 7231–7239 (2011).

[b13] Fan J. C., Shi Z. X., Lian M., Li H. & Yin J. Mechanically strong graphene oxide/sodium alginate/polyacrylamide nanocomposite hydrogel with improved dye adsorption capacity. J. Mater. Chem. A 1, 7433–7443 (2013).

[b14] Liu J. Q. . Synthesis of graphene peroxide and its application in fabricating super extensible and highly resilient nanocomposite hydrogels. ACS Nano 6, 8194–8202 (2012). 22917015 10.1021/nn302874v

[b15] Liu J. Q., Song G. S., He C. C. & Wang H. L. Self-healing in tough graphene oxide composite hydrogels. Macromol. Rapid. Commun. 34, 1002–1007 (2013). 23653331 10.1002/marc.201300242

[b16] Hu Z. Q. & Chen G. M. Novel nanocomposite hydrogels consisting of layered double hydroxide with ultrahigh tensibility and hierarchical porous structure at low inorganic content. Adv. Mater. 26, 5950–5956 (2014). 24923256 10.1002/adma.201400179

[b17] Liu M. . An anisotropic hydrogel with electrostatic repulsion between cofacially aligned nanosheets. Nature 517, 68–72 (2015). 25557713 10.1038/nature14060

[b18] Xia L.-W. . Nano-structured smart hydrogels with rapid response and high elasticity. Nat. Commun. 4, 2226 (2013). 23900497 10.1038/ncomms3226 PMC3731657

[b19] Salvadori B. & Dei L. Synthesis of Ca(OH)_2_ nanoparticles from diols. Langmuir 17, 2371–2374 (2001).

[b20] Nanni A. & Dei L. G. Ca(OH)_2_ nanoparticles from W/O microemulsions. Langmuir 19, 933–938 (2003).

[b21] Li Z. in Advanced concrete technology Wiley (2011).

[b22] Sun J.-Y. . Highly stretchable and tough hydrogels. Nature 489, 133–136 (2012). 22955625 10.1038/nature11409 PMC3642868

