# Supplementary material for: Super stretchable hydrogel achieved by non-aggregated spherulites with diameters <5 nm
Source: Nat Commun. 2016 Jun 29;7:12095. doi: 10.1038/ncomms12095 (PMC4931597; doi:10.1038/ncomms12095)
Supplement: Supplementary Information — Supplementary Figures 1-4 and Supplementary Table 1 [file ncomms12095-s1.pdf]

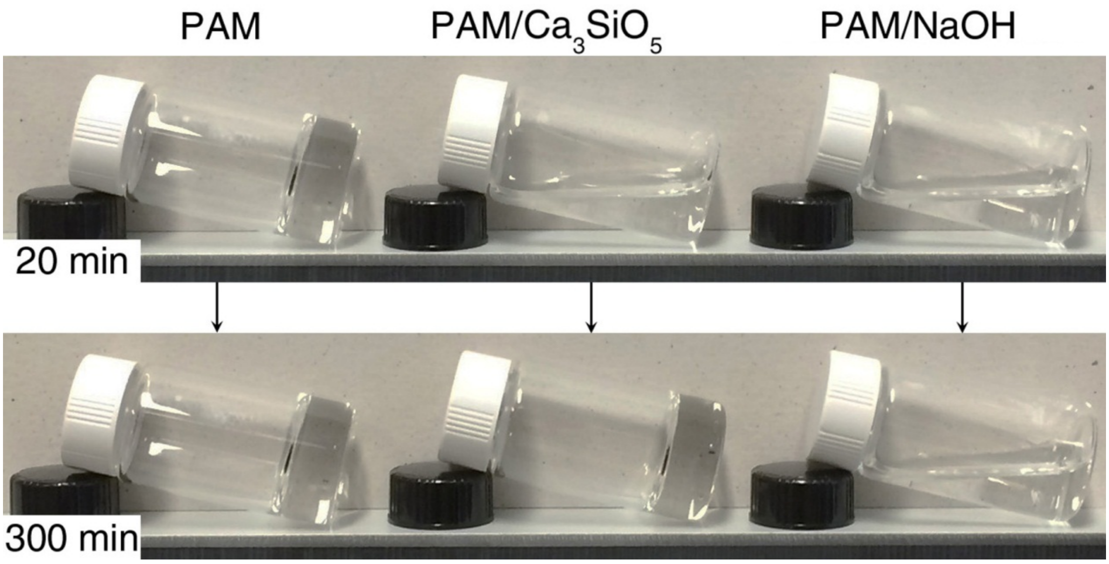

Supplementary Figure 1. Gelation behavior of PAM with the presence of tricalcium silicate (Ca_3SiO_5) and sodium hydroxide (NaOH) at room temperature. The polymer concentration was fixed at 20 wt%. The gelation time of PAM was prolonged from 20 min to 300 min with addition of 500 ppm tricalcium silicate, and gelation did not occur with addition of 500 ppm sodium hydroxide, indicating the inhibiting effect of OH^- on the initiation of polymerization.

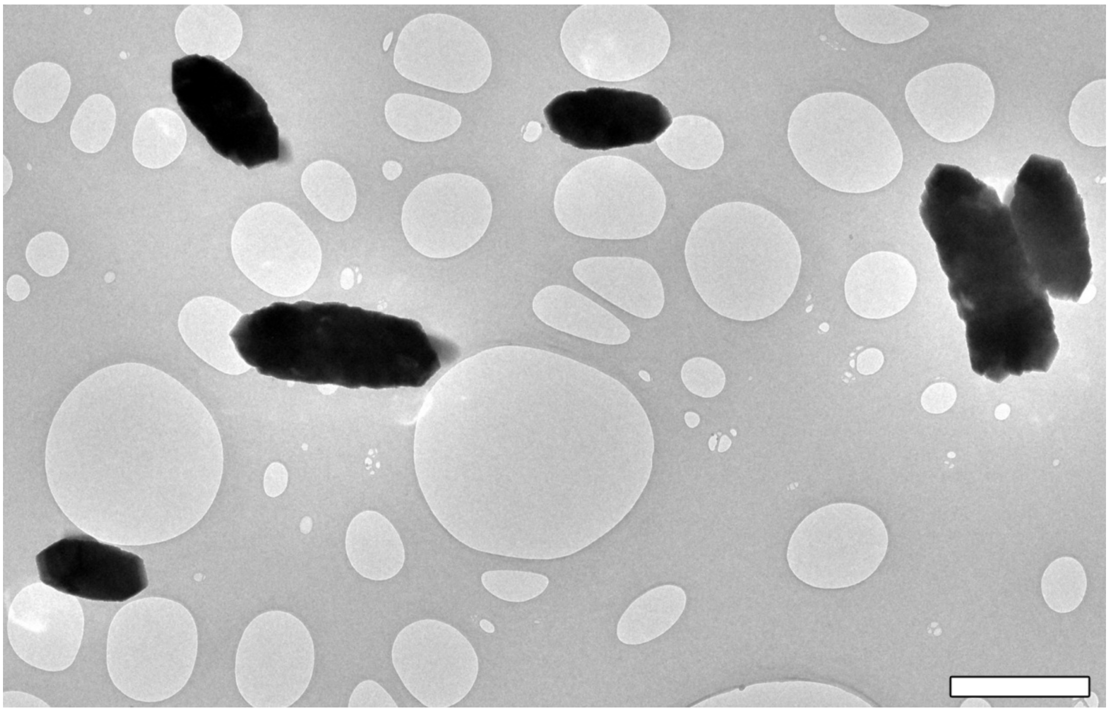

Supplementary Figure 2. A TEM image of calcium hydroxide micrometre crystals fabricated by cooling a suspension of calcium hydroxide at a concentration of 200 ppm from room temperature to 0 °C. Scale bar, 1 μm. In the figure, the black particles are calcium hydroxide micrometre crystals.

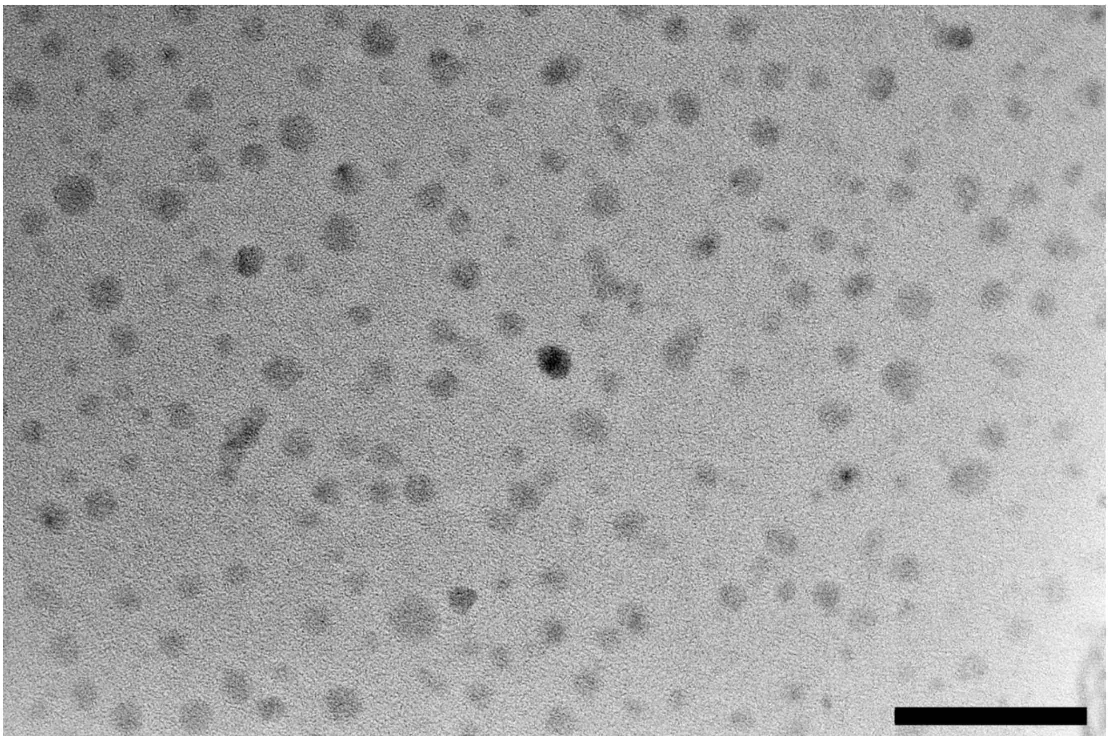

Supplementary Figure 3. A TEM image of CNS fabricated by maintaining a suspension of Portland cement at a concentration of 500 ppm at 0 °C for 3 days. Scale bar, 30 nm.

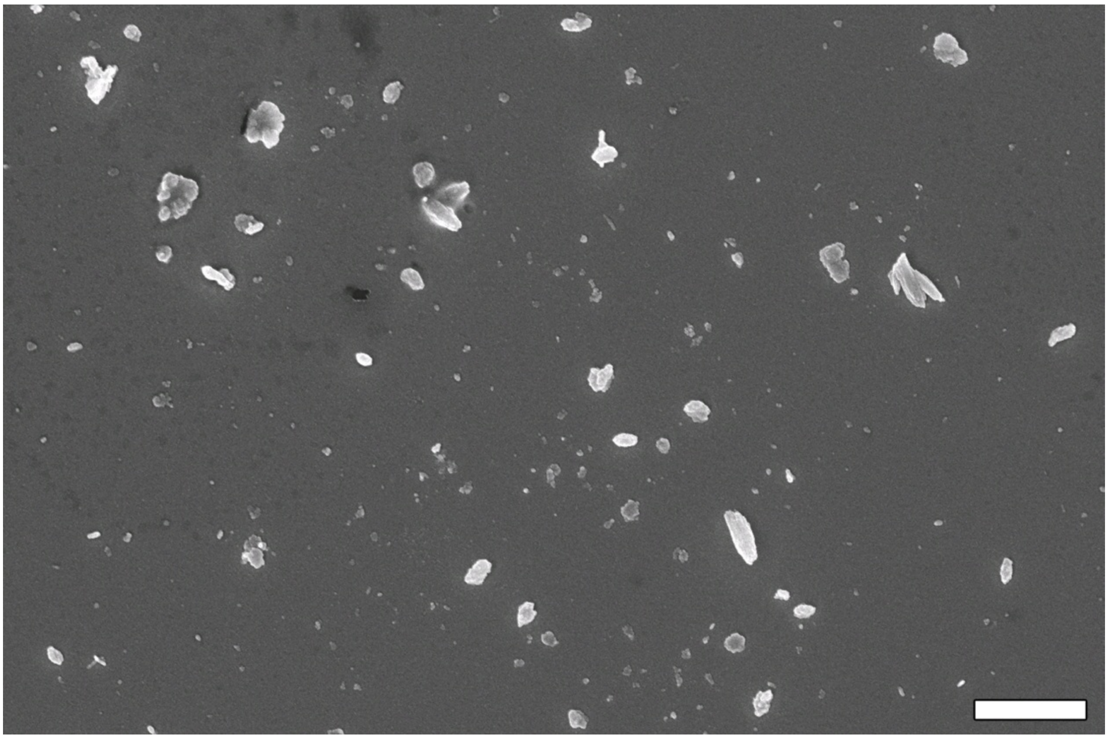

Supplementary Figure 4. A SEM image of tricalcium silicate dispersed by ethanol at a concentration of 500 ppm. Scale bar, 1 μm.

Supplementary Table 1. Comparison of size for crosslinking nanoparticles. At the same volume content and dispensability, the particle number density of CNS with diameters less than 5 nm is about 1000, 2000, 200, 5.0×10^5, and 3.8×10^6 times higher than that of clay, GO, LDH, TiNS and calcium hydroxide micro crystal, respectively.

Particle	Shape	Size (nm)	Functional surface area (nm^2)	Volume (nm^3)
clay (Hectorite)	rectangular plate	1×80×800	6.4×10^4	6.4×10^4
graphene oxide (GO)	cylinder	diameter=370<br>thickness=1.3<br>(for single layer)	1.1×10^5	1.4×10^5
layered double hydroxide (LDH)	cylinder	diameter=60<br>thickness=5<br>(for thorough exfoliation)	2.8×10^3	1.4×10^4
titanate(IV) nanosheet (TiNS)	cylinder	diameter=7500<br>thickness=0.75	4.4×10^7	3.3×10^7
calcium hydroxide micro crystal (Supplementary Fig. S2)	cylinder	diameter=500<br>thickness=1250	2.0×10^5	2.5×10^8
CNS	spherulite	diameter less than 5 nm	78.5	65.4
